# ‘If you have a problem with your heart, you have a problem with your life’: Self-perception and behaviour in relation to the risk of ischaemic heart disease in people living with HIV

**DOI:** 10.4102/phcfm.v7i1.772

**Published:** 2015-03-31

**Authors:** Ronel Roos, Hellen Myezwa, Helena van Aswegen

**Affiliations:** 1 Department of Physiotherapy, University of the Witwatersrand, South Africa

## Abstract

**Background:**

Ischaemic heart disease (IHD) is a global health problem and specifically relevant in the African context, as the presence of risk factors for IHD is increasing. People living with the human immunodeficiency virus (HIV)/acquired immune deficiency syndrome (AIDS) (PLWHA) are at increased risk for IHD due to increased longevity, treatment-specific causes and viral effects.

**Aim:**

To determine the self-perception and behaviour in relation to risk for IHD in a cohort of South African PLWHA.

**Methods:**

A qualitative study using semi-structured interviews with a card-sort technique was used to gather data from 30 individuals at an HIV clinic in Johannesburg. Descriptive analysis and conventional content analysis were done to generate the findings.

**Results:**

The median age of the cohort was 36.5 (31.8–45.0) years and they were mostly women (*n* = 25; 83.3%) who were employed (*n* = 17; 56.7%) and supporting dependents (*n* = 26; 86.7%). Fifteen (50%) participants did not perceive themselves at risk of IHD and reported having adequate coping behaviour, living a healthy lifestyle and being healthy since initiating therapy. Twelve (40%) did feel at risk because they experienced physical symptoms and had poor behaviour. Knowledge and understanding related to IHD, insight into own risk for IHD and health character in a context of HIV infection were three themes.

**Conclusion:**

This study highlights that participants did not perceive themselves to be at risk of IHD due to their HIV status or antiretroviral management. Education strategies are required in PLWHA to inform their personal risk perception for IHD.

## Abstrait

**‘Si vous avez un problème cardiaque, vous avez un problème de vie’: La perception de soi et le comportement par rapport au risque de maladie cardiaque ischémique chez les patients atteints du VIH.**

**Contexte:**

Les maladies cardiaques ischémiques (IHD) sont un problème de santé mondial particulièrement pertinent dans le contexte africain étant donné que la présence de facteurs de risques d'IHD augmente en raison de la présence élevée du virus humain de déficience immunitaire (HIV). Les personnes vivant avec le VIH ont un risqué accru de IHD dû àl'augmentation de la longévité, àdes causes spécifiques du traitement et àdes effets viraux. Cette étude a déterminé la perception de soi et le comportement par rapport au risque d'IHD chez un groupe de Sud-africains atteints du VIH. Un plan d’é tude qualitatif par le biais d'entrevues semi-structurées avec une technique de sélection des cartes a été utilisé pour collecter les données de trente individus àune clinique VIH àJohannesburg, en Afrique du Sud. Une analyse descriptive et une approche inductive avec une analyse conventionnelle du contenu ont été utilisées pour obtenir les résultats.

**Résultats:**

L’â ge moyen du groupe était de 36.5 (31.8–45.0) ans et comprenait surtout de femmes (*n* = 25; 83.3%) qui avaient un emploi (*n* = 17; 56.7%) et des personnes àcharge (*n* = 26; 86.7%). Quinze des participants (50%) ne se considéraient pas exposés au risque d'IHD car ils savaient faire face àla situation, en vivant sainement et en étant en bonne santé depuis qu'ils avaient commencé une thérapie antirétrovirale très active. Douze des participants (40%) se sentaient vulnérables car ils avaient ressenti des symptômes physiques et avaient un mauvais comportement dû àun mauvais régime alimentaire, des taux élevés de stress et le manque d'exercice. Trois des participants (10%) n’é taient pas sûrs de leur risque d'IHD. Les connaissances et la compréhension concernant l'IHD, la conscience de leurs propres risques d'IHD et le caractère sanitaire dans le contexte du VIH ont été identifiés comme les trois thèmes principaux se dégageant des données.

**Conclusion:**

Cette étude montre que les participants ne se considèrent pas en danger d'IHD en raison de leur statut VIH ou de leur traitement antirétroviral. Il faut éduquer les gens atteints du VIH pour influencer leur perception personnelle de risque d'IHD ce qui en retour pourrait amener des changements de comportement et la prévention d'IHD.

## Introduction

Ischaemic heart disease (IHD) is a global health concern, and this condition combined with stroke accounted for 12.9 million deaths globally in 2010.^1^ The greatest burden associated with IHD occurs in developing countries due to epidemiological transition: increased longevity of individuals, urbanisation and lifestyle changes.^2,3^ In sub-Saharan Africa IHD is a concern due to risk factors for IHD such as hypertension, diabetes, overweight and obesity, physical inactivity and dyslipidaemia.^4,5,6^ Additionally, the high prevalence of infection with the human immunodeficiency virus (HIV) augments the potential burden of IHD into the future.

Studies have shown that people living with HIV/acquired immune deficiency syndrome (AIDS) (PLWHA) are at an increased risk for developing IHD compared to non-infected individuals.^7,8^ Increased risk can be attributed to the presence of traditional risk factors, for example, smoking prevalence,^8^ metabolic effects of highly active antiretroviral therapy (HAART)^9,10^ and specific viral effects (e.g. increased inflammation^11,12^). Little research has been published regarding PLWHA's perception of IHD risk. Cioe, Crawford and Stein^13^ indicated that knowledge of IHD risk factors was fairly high, but not predictive of individuals’ perceived risk for IHD in the United States of America (USA). Additionally, the authors found that individuals’ risk perception was inaccurate when compared to their actual risk as determined by the Framingham Risk Score.

### Aim and objective

The aim of this study was to determine self-perception and behaviour in relation to the risk of IHD in a cohort of South African PLWHA. Considering the potential burden of IHD in sub-Saharan Africa, it is prudent to determine if PLWHA in Africa perceive themselves as at risk for IHD. This information may inform clinical practice and enable tailored education programmes.

## Research methods and design

### Study design

A qualitative study design was chosen to achieve the aims of the study. Qualitative inquiry provides the ideal means to describe the complex nature of humans and how individuals perceive their own experiences within a specific social context.^14^

### Study population

Participants were purposefully sampled according to the following inclusion criteria: 20–65 years of age, on HAART treatment for 6–12 months and ambulatory without an assistive device. Participants with a past medical history of cardiovascular disease, complaints of difficulty walking at the time of recruitment, pregnancy, complaints of acute illness or active opportunistic infection were excluded. The sample size was dependent upon information gathered during the interview process, until the point of saturation was reached.

### Assessment procedure

Participants completed a demographic questionnaire to capture their social and HIV background. Pharmacological treatment and details of specific HAART medication were collected from the clinic files. Participants’ latest CD4 count and viral load values were collected from the clinic laboratory database and clinic files.

Interviews were conducted in a private room using a semi-structured interview questionnaire. Open-ended questions related to living a healthy lifestyle and IHD were asked, with secondary prompting questions added. The card-sort technique was used to assist in generating information; this is associated with a constructivist approach, specifically Kelly's Personal Construct Theory: ‘they assume that people make sense of the world by categorising it, and that people can describe their own categorisation of the world with reasonable validity and reliability’.^15^ Card sort elicits individual understanding about topics and their relationships with each other. It is systematic and easy to use for both respondents and researchers.^15,16^

For the purposes of this study, a closed card-sort technique was used. Twenty three cards consisting of key phrases (English and isiZulu translation) with supporting pictures related to components of living a healthy lifestyle were utilised. An additional blank card was included that enabled each participant an opportunity to add an aspect of living a healthy lifestyle that was not presented on the other cards. Participants were asked to review the cards and sort them, from most important to least important on a continuum according to hierarchy of living a healthy lifestyle. Participants had freedom to overlap cards into piles on the line if the cards had similar importance. Their card selections were discussed and led to the open-ended questions related to IHD. All interviews were tape-recorded to allow for verbatim transcription at a later stage.

### Data analysis

An inductive approach to data analysis was followed using conventional content analysis.^17^ As stated by Thomas^18^: ‘inductive analysis refers to approaches that primarily use detailed readings of raw data to derive concepts, themes or a model through interpretation made from the raw data by an evaluator or researcher’. All interviews were transcribed verbatim from beginning to end in Microsoft Word. Codes and keywords were identified vertically and then for each question horizontally in all transcribed documentation. The frequencies on how often codes appeared were analysed question by question as a means of highlighting their importance. The frequency of similar codes contributed to data saturation. This was supported by the percentage of participants who had concepts in similar categories.

To reduce the data, the codes were collapsed into subcategories, then categories and lastly themes.^19^ Explanatory qualitative quotations provide descriptive data as per participants’ contextual explanations. Demographic information was analysed using SPSS IBM 21. Categorical data are presented as frequencies and percentages. Numerical data are expressed as medians (interquartile range).

### Ethical considerations

The study was approved by the University of the Witwatersrand Human Research Ethics Committee, permission was received from the clinic where the study was undertaken, and participants provided informed consent.

Triangulation strategies to improve trustworthiness of research findings included member checks of transcribed interviews by a senior qualitative researcher; a second researcher analysed 20% of transcribed interviews; 20% of study participants returned for a second appointment to review transcribed interviews and clarify key findings; and two focus group discussions with the researcher and three senior qualitative researchers were held to assist in identifying themes. A transparent coding process and inter-coder verification added to dependability of the research findings.

## Results

### Demographic characteristics

The median age of participants was 36.5 (31.8–45.0) years with 10.5 (7.0−12.0) months being the median time spent on HAART (Table 1). Most participants were diagnosed as having HIV infection between 2009 and 2010 (*n* = 21; 70%), and they were on the HAART regimen consisting of lamivudine, efavirenz and stavudine (*n* = 13; 43.3%) (Table 1). The majority of participants were female (*n* = 25; 83.3%), employed (*n* = 17; 56.7%), with a secondary school education (*n* = 16; 53.3%), and were supporting dependents (*n* = 26; 86.7%) (Table 1). Most perceived their health status as good (*n* = 17; 56.7%).

**TABLE 1:** Demographic characteristics of study participants (*N* = 30).

Variable	*N*	Median (interquartile range)	Variable	
			*n*	%
Age (years)	30	36.5 (31.8–45.0)	-	-
**Gender**	30	-		
Male	-	-	5	16.7
Female	-	-	25	83.3
Time on HAART (months)	30	10.5 (7.0–12.0)	-	-
CD4 count (cells/mm^3^)	30	282 (211.0–357.5)	-	-
Viral load (copies/mL)	28*	22.5 (0.0–51.3)	-	-
**Diagnosed with HIV infection**				
Prior to 2005	-	-	3	10.0
Between 2005 to 2008	-	-	6	20.0
Between 2009 to 2010	-	-	21	70.0
**HAART categories**				
Lamivudine, efavirenz, tenofovir	-	-	10	33.3
Lamivudine, efavirenz, stavudine	-	-	13	43.3
Other	-	-	7	23.3
**Educational level**				
No education	-	-	1	3.3
Primary school	-	-	5	16.7
education	-	-		
Secondary school education	-	-	16	53.3
Post-secondary school education	-	-	8	26.7
**Employment status**				
Unemployed	-	-	12	40.0
Employed	-	-	17	56.7
Self-employed	-	-	1	3.3
**Participants who had dependents**				
No	-	-	4	13.3
Yes	-	-	26	86.7
**Perception of health status**				
Average	-	-	8	26.7
Good	-	-	17	56.7
Excellent	-	-	5	16.7

*, Viral load findings only available for 28 participants.

*N* = 30.

### Self-perception and behaviour in relation to the risk of Ischaemic heart disease

Fifty percent of the participants (*n* = 15) did not perceive themselves as at risk for IHD; 12 (40%) did and three (10%) were unsure. Twenty six individuals (87%) thought one can prevent IHD from occurring in one's life. Two (6.6%) said it was unavoidable and two (6.6%) additional individuals were unsure if it was preventable.

Three prominent themes were identified during the data analysis: knowledge and understanding related to IHD, insight into their own risk, and health character in a context of HIV.

### Knowledge and understanding related to Ischaemic heart disease

Participants demonstrated knowledge and understanding related to the causes of IHD. Psychological factors such as stress and thinking too much were codes that presented often (*n* = 20; 66%). One participant stated this as follows:

‘Sometimes I wonder if it is not due to stress overload. The heart is always pumping fast and it can't relax. ’ (Participant 21, female, 49 years, no education and employed)

Not accepting one's HIV status (*n* = 1; 3%) and keeping problems to themselves (*n* = 3; 10%) to a lesser extent were noted to add to the risk for IHD:

‘When one has a lot of problems that you can't solve on your own, that you keep on yourself inside, that can cause heart disease. ’ (Participant 6, female, 39 years, secondary school education and employed)

The second most prominent cause of IHD reported by participants was related to nutrition, as reflected by specifics on diet (*n* = 16; 53%). This was voiced as follows:

‘I think eating habits, you need to feed your body just like you feed your mind, and you need to select what you eat. ’ (Participant 1, female, 36 years, post-secondary school education and employed)

Thirdly, participants explained the cause of IHD in relation to impaired structure and function of the heart. Understanding regarding the link between diet and altered function of the heart was also demonstrated:

‘When you are eating too much fat, the fat clots the heart. Pumping of the heart is not good. When it is very bad your heart can fail. ’ (Participant 11, male, 25 years, secondary school education and employed)

Smoking (*n* = 4; 13%) and not being active (*n* = 2; 7%) as causative factors for IHD were codes but did not appear often. Participants demonstrated knowledge and understanding related to the consequences of IHD by highlighting signs and symptoms, morbidity and mortality aspects related to IHD. Chest pain (*n* = 13; 43%) and breathing difficulties (*n* = 11; 37%) were issues most often highlighted. Illustrative quotes of signs and symptoms of IHD were:

‘I think swelling because the circulation is not well. Sweating and breathing problems and chest pains most of the time. ’ (Participant 8, female, 28 years, post-secondary school education and employed)

Participants knew that an increased risk for mortality was linked with IHD (*n* = 8; 27%):

‘If someone is having heart disease they can die from heart disease or if they take care of themselves they can live long. ’ (Participant 29, female, 32 years, secondary school education and employed)

Lastly, knowledge and understanding regarding prevention strategies for IHD in the form of lifestyle change, coping behaviour, health-seeking behaviour and medical management were reported. These facets were explained:

‘Yes, if you do all the right things like not eating fatty food, not using cooking oil, if you don't smoke, if you do your exercises and eat your vegetables and fruit. Healthy balanced life; drink enough water, sleep enough and don't stress too much. You always make sure that you are in the middle and level. ’ (Participant 29, female, 32 years, secondary school education and employed)

Some participants stated that outside assistance was needed to prevent IHD:

‘Yes, they must come to the clinic at the hospital to get treatment. Only the hospital can make the heart disease right … A person can't help themselves; they can't do something, the hospital must help. ’ (Participant 26, female, 45 years, primary school education and employed)

Another reported:

‘Yes you can … like an operation to your heart. I don't know anything else that will help. ’ (Participant 10, female, 26 years, secondary school education and unemployed)

### Insight into own risk for Ischaemic heart disease

Participants who perceived themselves not at risk for IHD explained that this was due to following a healthy lifestyle, specific coping behaviours and maintaining health since using HAART. One participant voiced it as follows:

‘No, at the moment with HIV and the life I have been leading before, it has been a healthy life throughout and I have never thought that I could get heart disease. But in the course of time you are no longer as young and fit as you used to be, you can come across a lot of problems and could get heart disease, but at the moment I don't think so. ’ (Participant 28, male, 49 years, secondary school education and employed)

Another stated:

‘No because something that can cause a painful heart I try and take away or leave alone. ’ (Participant 21, female, 49 years, no education and employed)

Antiretroviral therapy was seen as a protective factor against IHD:

‘No since I am taking ARVs I am healthy. ’ (Participant 14, female, 32 years, secondary school education and unemployed)

In contrast, participants who perceived themselves to be at an increased risk for IHD explained that it was due to their behaviour and/or experiencing physical symptoms:

‘Sometimes, because of the stress and unhealthy diet and not exercising. I think sometimes I can feel my heart tension that is not normal. Maybe you feel that you are stressed and angry. Anger causes your high blood to go higher and you can even feel that your heart is not “*klopping*” [*beating*] normal. You feel that it is beating faster. It feels like you can hold it, then you feel the short breath and everything. ’ (Participant 3, female, 36 years, secondary school education and employed)

Another stated:

‘I think so because when I am worried there is pain straight in the heart. Sometimes I feel weak and the pain goes to my back. ’ (Participant 7, male, 45 years, post-secondary school education and unemployed)

### Health character in a context of HIV infection

Participants demonstrated knowledge and understanding regarding why it is important to adhere to HAART. One participant stated:

‘To live longer, that is why I am taking ARVs so that I live longer for my children. I know that the ARVs are helping me to keep the sickness down … ’ (Participant 6, female, 39 years, secondary school education and employed)

Participants had knowledge of comorbidities that might develop during the course of their illness:

‘TB, losing weight, skin can change, some people gets a rash or get sores in the mouth. ’ (Participant 22, male, age 34 years, primary school education and unemployed)

Coping was deemed an important facet that influenced participants’ health character and coping beliefs. Certain coping behaviours were identified as ways in which participants attempted to improve their lives. One participant explained:

‘It is important to have support from my family especially my mother, because in many ways she supported me in my life; if I am sick, she was there for me. ’ (Participant 10, 26 years, secondary school education and unemployed)

Being able to live a healthy life was often hindered by environmental factors such as unemployment. One participant explained:

‘To get a job, that is what makes me stressed. Not having a job. ’ (Participant 19, female, 48 years, post-secondary school education and unemployed)

The importance of having employment to ensure an adequate diet was emphasised:

‘Yes, it is important to have a job so that you will be able to buy food to be able to take your medication. ’ (Participant 25, female, 31 years, primary school education and unemployed)

Taking responsibility for health and self-regulating behaviour even in challenging circumstances were important components for functioning with HIV infection. This self-regulation was especially noted when participants’ discussed how they used HAART (*n* = 17; 56.7%):

‘I think to take my medication at the right time. Not to take my medication at any time. If I take my medication at eight o'clock I need to take my medication at the same time. It is very important and healthy. ’ (Participant 14, female, 32 years, secondary school education and unemployed)

Another said:

‘You must eat first then take your medication like ARVs. You can't drink it on an empty stomach. ’ (Participant 10, female, 26 years, secondary school education and unemployed)

## Discussion

In a South African context prevalence of HIV infection remains disproportionally high in females in comparison to males.^20^ More women access HAART in sub-Saharan Africa^21^ and in South Africa.^22^ The international literature suggests that individuals often underestimate their risk for IHD,^23,24^ and this is identified more often in women.^25,26^ So it was important to evaluate self-perception and behaviour in relation to the risk of IHD in a South African cohort of PLWHA.

Participants had some knowledge and understanding of IHD, and the majority of participants had a secondary educational level. These two factors might explain why 12 participants (40%) perceived themselves at risk for IHD. The literature suggests that one's knowledge of risk factors for IHD influences one's self-perception.^23^ Potvin, Richard and Edwards^27^ report that individuals are more likely to know behavioural compared to physiological risk factors of IHD. Out of a sample of 23 129 Canadian participants, 60% reported fat in food, 52% smoking, and 41% lack of exercise as risk factors for IHD. Only 32% reported excess weight, 27% elevated cholesterol and 22% high blood pressure. The education level of participants was strongly associated with participants’ ability to recall risk factors of IHD.^27^

Ansa, Oyo-Ita and Essien,^28^ who investigated knowledge of risk factors for IHD in university staff in Nigeria, reported smoking (70.6%), use of alcohol (52.8%), obesity (41.6%), sedentary lifestyle (16.6%) and oral contraceptive use (6.4%) as risk factors named. Senior staff with more education were more likely than junior staff to report all of the risk factors. Pace et al.^29^ reported an interesting finding regarding education and IHD, in that individuals with a secondary school education or less were likely to report that they know about heart disease but that it was not a concern to them.

In contrast Cioe et al.^13^ found a low, non-significant relationship between risk factor knowledge (Heart Disease Fact Questionnaire) and perceived risk (Perception of Risk of Heart Disease Scale) (*r* = 0.13; *p* ˃ 0.05). Cioe et al.'s study was conducted in PLWHA in Rhode Island, USA and consisted mostly of men (62.3%), whilst our study primarily includes women. Even though the authors found a low, non-significant relationship, 97% of their population knew that smoking and being overweight were risk factors for cardiovascular disease.

In our study smoking as a risk factor for IHD was voiced by four individuals (13%), but none voiced overweight or obesity as a risk factor for IHD. This is of concern, considering that obesity in PLWHA is becoming more prevalent.^30^ Hurley et al.^31^ found that when individuals are on a regimen containing stavudine a significant change (*p* < 0.001) in body mass index of 2.2 kg/m^2^ (95% CI 1.5–2.9) in women and 2.4 kg/m^2^ (95% CI 1.7–3.1) in men occurs during the first year following initiation. Ninety per cent of participants in Cioe et al.'s study^13^ also knew that a high cholesterol, high fat diet and lack of exercise contribute to an increased risk for IHD. Similarities exist between Cioe et al.'s and our study with regard to dietary contribution and lack of exercise as risk factors for IHD. Our study therefore highlights that education strategies are necessary to enlighten PLWHA living in South Africa about the impact of smoking and obesity on their potential future risk for IHD.

Participants in this study who perceived an increased risk for IHD explained that it was due to lifestyle risk factors such as stress and poor diet. The general public in South Africa are known to have higher levels of stress^32^ compared to individuals living in the USA.^33^ Participants in our study also had to cope with the stigma that surrounds HIV infection and the difficulties associated with employment and participation in the wider community. This risk factor was therefore highlighted as an important factor to address when implementing management strategies for IHD in PLWHA in South Africa.

Individual participants experienced symptoms such as chest pain, especially when they were under a lot of stress. A number of factors contribute to reasons why participants did not address their symptoms. Bodily sensations provide information to an individual, and depending on one's past experiences a person might or might not act on these experiences.^34^ Corbin^34^ states that individuals are likely to wait and see what happens, and if the sensations become more frightening they will seek medical advice.

The Shifting Perspectives Model of chronic illness described by Paterson^35^ could also be used to explain why participants did not seek medical advice. The Shifting Perspectives Model demonstrates that individuals living with chronic illness (e.g. PLWHA) continually shift between two states: illness-in-the-foreground or wellness-in-the-foreground. Wellness is determined by comparing the experience (e.g. chest pain) to what is known and understood about illness, and vice versa. Paterson^35^ explains that a perception of losing control is the major factor that will move an individual from a state of wellness-in-the-foreground to illness-in-the-foreground. It is therefore important to monitor subjective symptoms of IHD closely in PLWHA to identify individuals who require further investigations, as they might not see their symptoms as problematic when compared to previous illness experiences.

It was encouraging to note that participants understood the importance of adhering to their HAART regimens and followed advice given by the clinic on how to take their medication on a daily basis. Reda and Biadgilign^36^ reported that lack of adherence to HAART in Africa is due to financial constraints and poor food security. Individuals in our study voiced that being unemployed influenced their diet and created significant amounts of stress. Educational strategies would therefore not completely address individuals’ stress levels and dietary choices. Part of the wider response addressing social problems facing South Africa should include addressing unemployment and livelihood.

The following limitations should be acknowledged when interpreting the findings from the current study. This study used a qualitative approach and consisted of 30 individuals from one HIV clinic in an urban setting, which limits generalisation of findings to the larger South African and African HIV community. In addition, statistical conclusions from the quantitative data presented here can only be related to the current study population. Data saturation occurred in that the same codes did occur more frequently; however, those that did not were interpreted as knowledge gaps, as this was directed content analysis and participants gave information they knew about within the conceptual framework used. It should also be noted that self-perception and behaviour of participants were influenced by participants’ knowledge and understanding of IHD.

## Conclusion

Our study supports the need to develop educational programmes to improve IHD risk perception in PLWHA in Johannesburg, South Africa. Programmes are needed to educate and focus on the impact of advancing age, obesity, physical inactivity, HIV and HAART sequelae on risk for IHD.

Stress was highlighted as a significant risk factor for IHD by participants and often given as a reason why they perceived themselves to be at risk. Intervention strategies to assist individuals on how to manage their stress levels, such as exercise programmes, is key to reduce risk factors for IHD.

As the roll-out of HAART to PLWHA increases in South Africa, morbidity associated with non-communicable diseases such as IHD could increase, as has been shown internationally. Educating PLWHA about risk factors for IHD, screening for risk factors and implementing intervention strategies to change behaviour are important means to lessen the potential burden of IHD in PLWHA.

## References

[1] Lozano R, Naghavi M, Foreman K, et al Global and regional mortality from 235 causes of death for 20 age groups in 1990 and 2010: A systematic analysis for the Global Burden of Disease Study. Lancet. 2010;380(9859):2095–2128. http://dx.doi.org/10.1016/S0140-6736(12)61728-0 10.1016/S0140-6736(12)61728-0 PMC10790329 23245604

[2] Mensah GA Ischaemic heart disease in Africa. Heart. 2008; 94(7):836−843 http://dx.doi.org/10.1136/hrt.2007.136523 18552223 10.1136/hrt.2007.136523

[3] Gaziano TA Cardiovascular disease in the developing world and its cost-effective management. Circulation. 2005;112(23):3547–3553. http://dx.doi.org/10.1161/CIRCULATIONAHA.105.591792 10.1161/CIRCULATIONAHA.105.591792 16330695

[4] Onen CL Epidemiology of ischaemic heart disease in sub-Saharan Africa. Cardiovasc J Afr. 2013; 24(2):34–42. http://dx.doi.org/10.5830/CVJA-2012-071 23612951 10.5830/CVJA-2012-071 PMC3734874

[5] Danaei G, Finucane MM, Lin JK, et al National, regional, and global trends in systolic blood pressure since 1980: Systematic analysis of health examination surveys and epidemiological studies with 786 country-years and 5.4 million participants. Lancet. 2011;377:568–577. http://dx.doi.org/10.1016/S0140-6736(10)62036-3 10.1016/S0140-6736(10)62036-3 21295844

[6] Finucane MM, Stevens GA, Cowan MJ, et al National, regional, and global trends in body-mass index since 1980: Systematic analysis of health examination surveys and epidemiological studies with 960 country-years and 9.1 million participants. Lancet. 2011;377:557–567. http://dx.doi.org/10.1016/S0140-6736(10)62037-5 10.1016/S0140-6736(10)62037-5 PMC4472365 21295846

[7] Triant VA, Lee H, Hadigan C, Grinspoon SK Increased acute myocardial infarction rates and cardiovascular risk factors among patients with human immunodeficiency virus disease. J Clin Endocrinol Metab. 2007;92(7):2506–2512. http://dx.doi.org/10.1210/jc.2006-2190 17456578 10.1210/jc.2006-2190 PMC2763385

[8] Saves M, Chene G, Ducimetiere P, et al Risk factors for coronary heart disease in patients treated for human immunodeficiency virus infection compared with the general population. HIV/AIDS. 2003;37:292–298. http://dx.doi.org/10.1086/375844 10.1086/375844 12856222

[9] De Wit S, Sabin CA, Weber R, et al Incidence and risk factors for new-onset diabetes in HIV-infected patients: The DATA Collection on Adverse Events of Anti-HIV Drugs (D:A:D) Study. Diabetes Care 2008;31:1224–1229. http://dx.doi.org/10.2337/dc07-2013 10.2337/dc07-2013 PMC2746200 18268071

[10] Friis-Møller N, Weber R, Reiss P, et al Cardiovascular disease risk factors in HIV patients – association with antiretroviral therapy. Results from the DAD study. AIDS. 2003;17(8):1179–1193. http://dx.doi.org/10.1097/00002030-200305230-00010 12819520 10.1097/01.aids.0000060358.78202.c1

[11] De Luca A, De Gaetano Donati K, Colafigli M, et al The association of high-sensitivity C-reactive protein and other biomarkers with cardiovascular disease in patients treated for HIV: A nested case-control study. BMC Infect Dis. 2013;13(414):1–12. http://dx.doi.org/10.1186/1471-2334-13-414 10.1186/1471-2334-13-414 PMC3846422 24004495

[12] Triant VA, Meigs JB, Grinspoon SK Association of C-Reactive protein and HIV infection with acute myocardial infarction. J AIDS. 2009;51(3):268–273. http://dx.doi.org/10.1097/QAI.0b013e3181a9992c 10.1097/QAI.0b013e3181a9992c PMC2763381 19387353

[13] Cioe PA, Crawford SL, Stein MD Cardiovascular risk-factor knowledge and risk perception among HIV-infected adults. JANAC. 2013;25(1):60–69. 24070645 10.1016/j.jana.2013.07.006 PMC3875835

[14] Portney LG, Watkins MP Qualitative Research In: Cohen M, editor Foundations of Clinical Research; applications to practice, 3rd ed Pearson Education, Inc., New Jersey: Upper Saddle River, 2009; pp. 306–313.

[15] Rugg G, McGeorge P The sorting techniques: A tutorial paper on card sorts, picture sorts and item sorts. Expert Systems. 1997;14(2):80–93. http://dx.doi.org/10.1111/1468-0394.00045

[16] Fincher S, Tenenberg J Making sense of card sorting data. Expert Systems. 2005;22(3): 89–93. http://dx.doi.org/10.1111/j.1468-0394.2005.00299.x

[17] Hsieh HF, Shannon SE Three approaches to qualitative content analysis. Qual Health Res. 2005;15(9):1277–1288. http://dx.doi.org/10.1177/1049732305276687 16204405 10.1177/1049732305276687

[18] Thomas DR A general inductive approach for analysing qualitative evaluation data. Am J Eval. 2006;27(2):237–246. http://dx.doi.org/10.1177/1098214005283748

[19] Smith CP Content analysis and narrative analysis In: Reis T, Judd C, editors Handbook of research methods in social and personality psychology. New York: Cambridge University Press, 2000; pp. 313–326.

[20] Statistics South Africa Mid-year population release, 2013 Pretoria: Government of South Africa: 2013.

[21] Braitstein P, Boulle A, Nash D, et al Gender and the use of antiretroviral treatment in resource-constraint settings: Findings from a multicentre collaboration. J Womens Health. 2008;17(1):47–55. http://dx.doi.org/10.1089/jwh.2007.0353 10.1089/jwh.2007.0353 18240981

[22] Cornell M, Schomaker M, Garone DB, et al Gender differences in survival among adult patients starting antiretroviral therapy in South Africa: A multicentre cohort study. PLOS Med. 2012;9(9):e1001304 http://dx.doi.org/10.1371/journal.pmed.1001304 22973181 10.1371/journal.pmed.1001304 PMC3433409

[23] Meischke H, Sellers DE, Goff DC, et al Factors that influence personal perceptions of the risk of an acute myocardial infarction. Behav Med. 2000;26(1):4–13. http://dx.doi.org/10.1080/08964280009595748 10971879 10.1080/08964280009595748

[24] Avis NE, Smith KW, McKinlay JB Accuracy of perceptions of heart attack risk: What influences perceptions and can they be changed? AJPH. 1998;79(12):1608–1612. http://dx.doi.org/10.2105/AJPH.79.12.1608 10.2105/ajph.79.12.1608 PMC1349762 2817187

[25] Hart PL Women's perceptions of coronary heart disease: An integrative review. J Cardiovasc Nurs. 2005;20(3):170–176. http://dx.doi.org/10.1097/00005082-200505000-00008 15870587 10.1097/00005082-200505000-00008

[26] Legato MJ, Padus EP, Slaughter E Women's perceptions of their general health, with special reference to their risk of coronary artery disease: Results of a national telephone survey. J Womens Health. 1997;6(2):189–198. http://dx.doi.org/10.1089/jwh.1997.6.189 9140853 10.1089/jwh.1997.6.189

[27] Potvin L, Richard L, Edwards AC Knowledge of cardiovascular disease risk factors among the Canadian population: Relationships with indicators of socioeconomic status. CMAJ. 2000;162(9):S5–S11. 10813022 PMC1232442

[28] Ansa VO, Oyo-Ita A, Essien OE. Perception of ischaemic heart disease, knowledge of and attitude to reduction of its risk factors. EAMJ. 2007;84(7):318–323. 10.4314/eamj.v84i7.9586 17886425

[29] Pace R, Dawkins N, Wang B, et al Rural African Americans’ dietary knowledge, perceptions, and behaviour in relation to cardiovascular disease. Ethn Dis. 2008;18:6–12. 18447092

[30] Crum-Cianflone N, Roediger MP, Eberly L, et al Increasing rates of obesity among HIV-infected persons during the HIV epidemic. PLOS One. 2010;5(4):e10106 http://dx.doi.org/10.1371/journal.pone.0010106 20419086 10.1371/journal.pone.0010106 PMC2856157

[31] Hurley E, Coutsoudis A, Giddy J, Knight SE, Loots E, Esterhuizen TM Weight evolution and perceptions of adults living with HIV following initiation of antiretroviral therapy in a South African urban setting. SAMJ. 2011;101(9):645–650. 21920157

[32] Hamad R, Fernald LCH, Karlan DS, Zinman J Social and economic correlates of depressive symptoms and perceived stress in South African adults. JECH. 2008;62:538–544. http://dx.doi.org/10.1136/jech.2007.066191 10.1136/jech.2007.066191 18477753

[33] Cohen S, Janicki-Deverts D Who's stressed? Distributions of psychological stress in the United States in probability samples from 1983, 2006 and 2009. J Applied Soc Psychol. 2012;42(6):1320–1334. http://dx.doi.org/10.1111/j.1559-1816.2012.00900.x

[34] Corbin JM The body in health and illness. Qual Health Res. 2003;13(2):256–267. http://dx.doi.org/10.1177/1049732302239603 12643032 10.1177/1049732302239603

[35] Paterson BL The Shifting Perspectives Model of chronic illness. J Nurs Scholarsh. 2001;33(1):21–26. http://dx.doi.org/10.1111/j.1547-5069.2001.00021.x 11253576 10.1111/j.1547-5069.2001.00021.x

[36] Reda AA, Biadgilign S Determinants of adherence to antiretroviral therapy among HIV-infected patients in Africa. AIDS Res Treat. 2012:574656 http://dx.doi.org/10.1155/2012/574656 22461980 10.1155/2012/574656 PMC3296173

